# Supplementary material for: Intrinsic lipid binding activity of ATG16L1 supports efficient membrane anchoring and autophagy
Source: EMBO J. 2019 Apr 1;38(9):e100554. doi: 10.15252/embj.2018100554 (PMC6484409; doi:10.15252/embj.2018100554)
Supplement: Supplementary file 1 — Expanded View Figures PDF [file EMBJ-38-e100554-s001.pdf]

Expanded View Figures

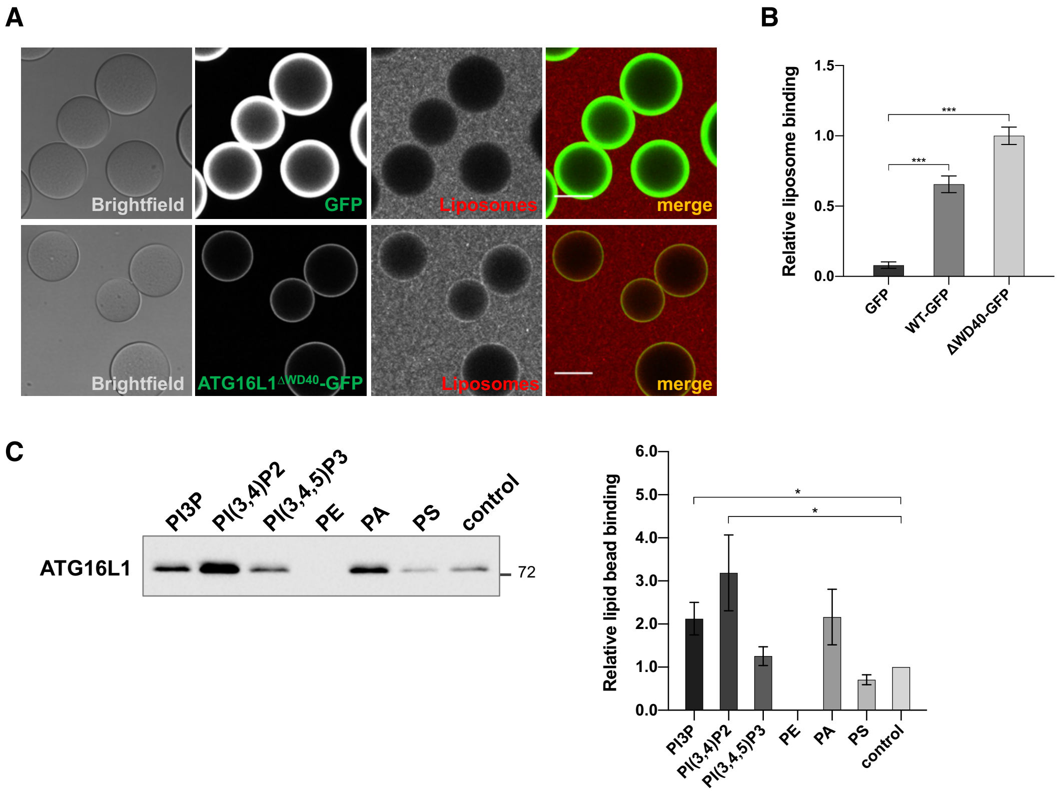

Figure EV1. Further characterisation of ATG16L1 lipid binding activity.

A Microscopy-based protein–liposome binding assay. ATG16L1$^{\Delta WD40}$-GFP or GFP alone was purified from ATG5$^{-/-}$ cells and immobilised on beads followed by incubation in the presence of rhodamine-labelled liposome preparations containing PI3P. Scale bar: 50 μm.

B Quantification of relative liposome binding in (A) along with ATG16L1^{WT}-GFP from Fig 3E. Quantifications depict means and error bars (SEM) from three independent experiments. ***$P \leq 0.001$ (pairwise unpaired Student's *t*-test).

C Lipid binding experiment using the indicated lipid-coated beads incubated with recombinant wild-type ATG16L1 purified from insect cells. Bound proteins were analysed by Western blot using anti-ATG16L1 antibodies. Right panel shows quantifications depicting means and error bars (SEM) from at least three independent experiments. *$P \leq 0.05$ (pairwise unpaired Student's *t*-test).

© 2019 The Authors

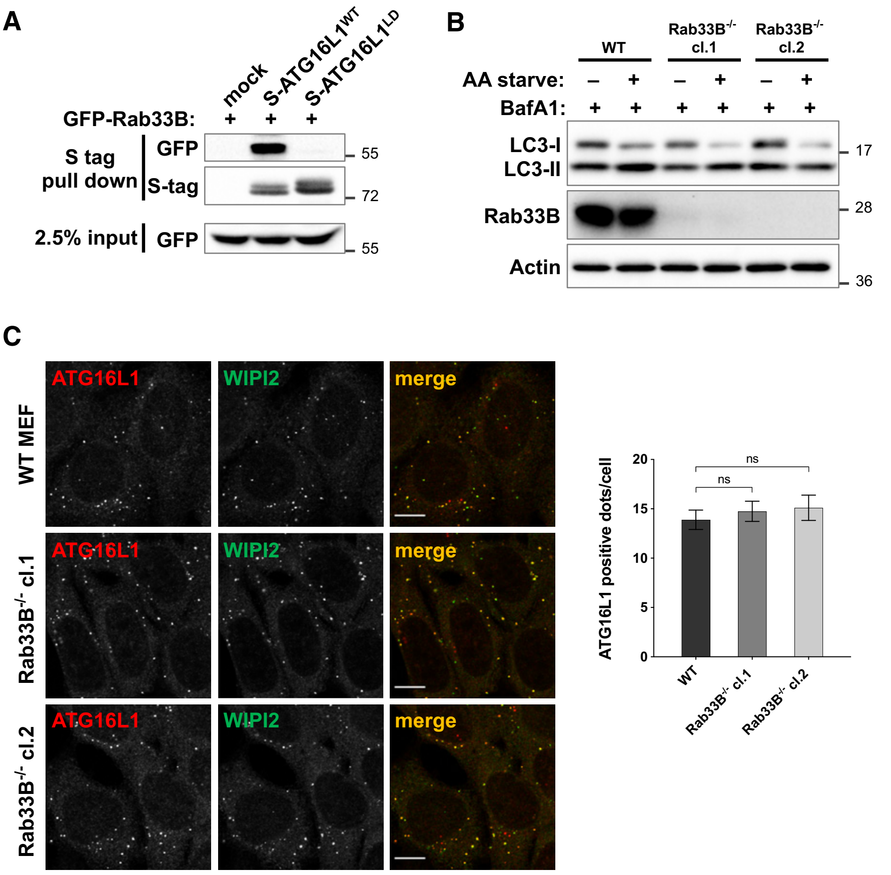

Figure EV2. Binding of Rab33B to ATG16L1^{WT} and autophagy assays in Rab33B$^{-/-}$ cells.

A Protein–protein interaction assay in 293T cells transiently transfected with the indicated Flag-S-tagged ATG16L1 constructs and GFP-Rab33B. S tag pull-down was performed, and protein complexes were analysed by immunoblotting using the indicated antibodies.

B Wild-type MEFs or Rab33B$^{-/-}$ clones were amino acid starved (AA starve) to induce autophagy in the presence of BafA1 for 2 h prior to lysis. Cell lysates were analysed using the indicated antibodies.

C Cells as in (B) were amino acid starved for 2 h prior to immunofluorescence analyses using antibodies against endogenous ATG16L1 (red) and WIPI2 (green). Quantification of ATG16L1 positive puncta is shown on the right panel depicting means and error bars (SEM) from at least three independent experiments. ns $P > 0.05$ (pairwise unpaired Student's t-test). Scale bar: 9 μm.

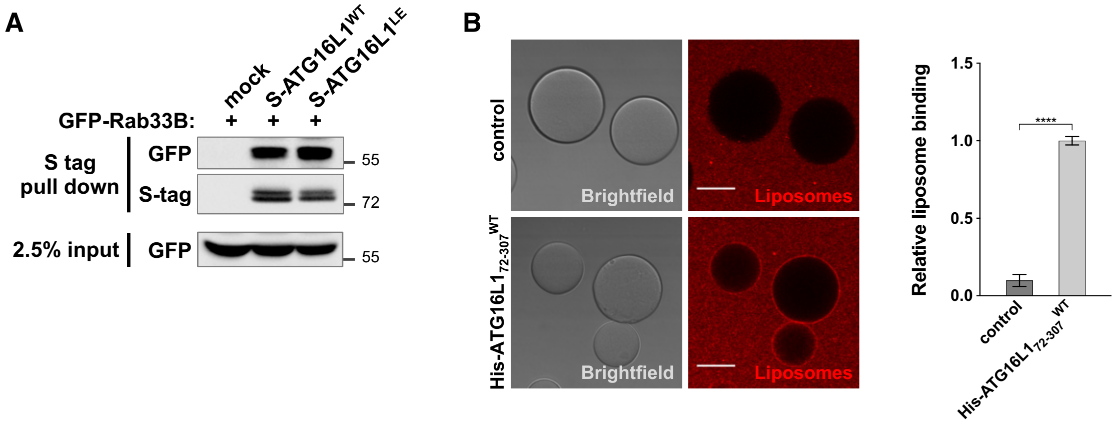

Figure EV3. Microscopy-based liposome binding assay using His-ATG16L1$_{72–307}$^{WT}.

A Protein–protein interaction assay in 293T cells transiently transfected with the indicated Flag-S-tagged ATG16L1 constructs and GFP-Rab33B. S tag pull-down was performed, and protein complexes were analysed by immunoblotting using the indicated antibodies.

B Microscopy-based protein–liposome binding assay. His-ATG16L1$_{72–307}$^{WT} immobilised on His beads or His-beads alone were incubated in the presence of rhodamine-labelled liposome preparations containing PI3P. Scale bar: 50 μm. The right panel shows quantification of relative liposome binding and depicts means and error bars (SEM) from at least three independent experiments. ****$P \leq 0.0001$ (pairwise unpaired Student's t-test).

 © 2019 The Authors

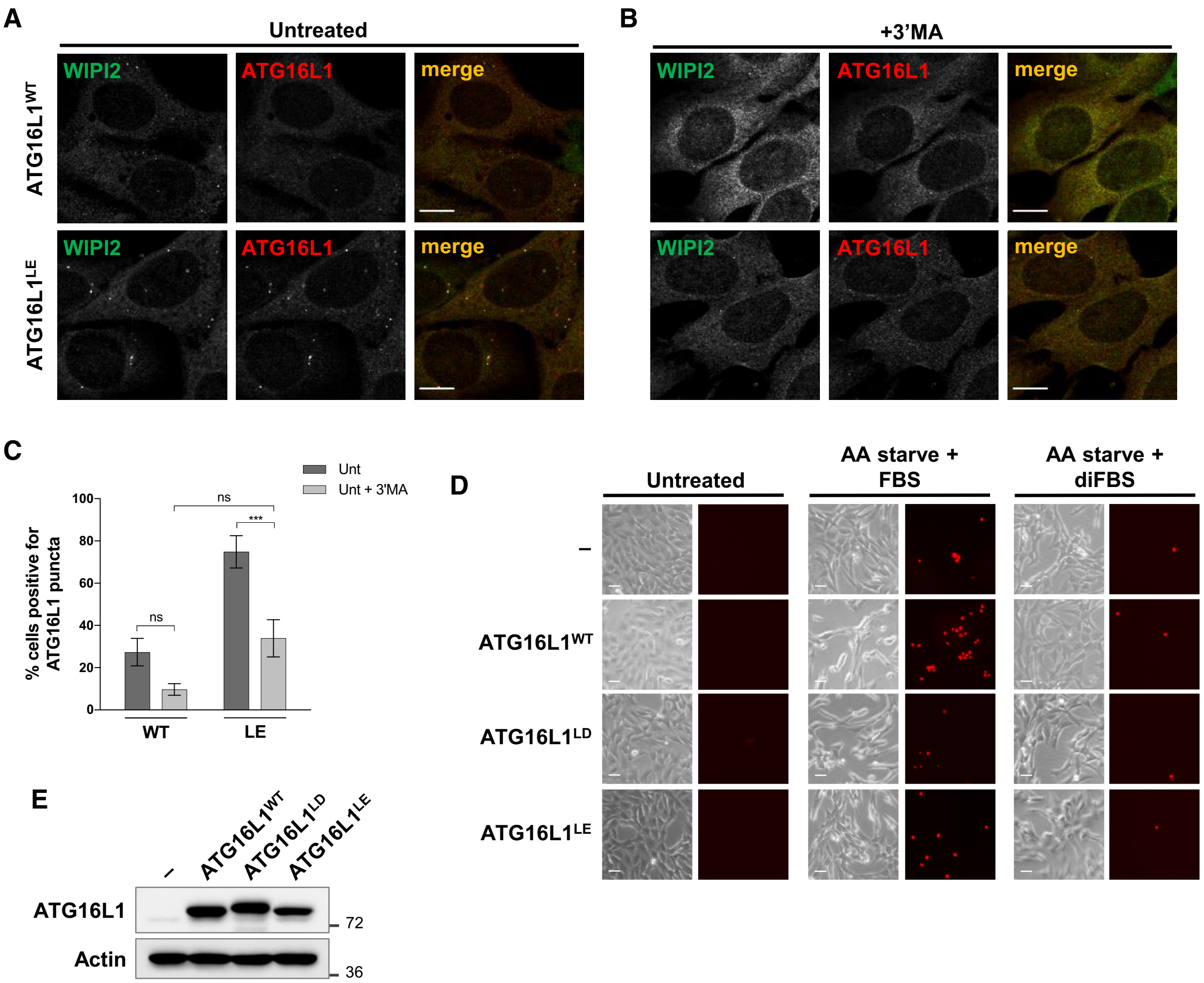

Figure EV4. Analyses of ATG16L1^{LE} puncta under full growth condition.

A ATG16L1$^{-/-}$ cells reconstituted with ATG16L1^{WT} or ATG16L1^{LE} were cultured in full growth media prior to immunofluorescence analyses using antibodies against WIPI2 (green) or ATG16L1 (red). Scale bar: 9 μm.

B Cells as in (A) with the addition of 3′MA treatment for 30 min. Scale bar: 9 μm.

C Quantification of percentage of cells positive for ATG16L1 puncta in (A) and (B). Quantifications depict means and error bars (SEM) from at least three independent experiments. ns $P > 0.05$, ***$P \leq 0.001$ (pairwise unpaired Student's *t*-test).

D Ferroptosis assay in ATG16L1$^{-/-}$ cells stably expressing F-S-tagged ATG16L1^{WT}, ATG16L1^{LD} or ATG16L1^{LE}. Cells were cultured in amino acid free media (AA starve) in the presence of 10% FBS or 10% dialysed FBS (diFBS) for 24 h. Representative PI staining and phase contrast images are shown (relevant to Fig 7G). Scale bar: 30 μm.

E Western blot analyses of ATG16L1$^{-/-}$ cells stably expressing F-S-tagged ATG16L1^{WT}, ATG16L1^{LD} or ATG16L1^{LE} using the indicated antibodies (relevant to D and Fig 7G).

© 2019 The Authors
